# The Effects of Multilayered Disorder Characteristics on Fear of Crime in Korea

**DOI:** 10.3390/ijerph17249174

**Published:** 2020-12-08

**Authors:** Su Jin Kang, Wonseok Seo

**Affiliations:** Department of Urban Planning and Real Estate, Chung-Ang University, Seoul 06974, Korea; sujin4644@naver.com

**Keywords:** fear of crime, mental health, physical disorder, social disorder, multilayered disorder, neighborhood, city

## Abstract

Fear of crime has a negative impact on the mental health of individuals, limiting their physical and social abilities. Moreover, the prevalence of such fear in the neighborhood weakens the bonds between neighbors and the overall community network, thereby undermining social capital and impeding the city’s sustainability. Disorder is a multilayered process shaped by type and spatial level and has a complex effect on fear of crime. Using a multilevel ordered logistic model, this study determines a more comprehensive cause of fear of crime by verifying the multilayered effects of disorder in Korea. The results include four main findings. First, people are relatively unaware of disorder occurring at the neighborhood level, and more sensitive to disorder at the city level. Second, social disorder has a more significant effect on fear of crime than physical disorder. Third, fear of crime is more affected by indirect factors than by direct factors and actual crimes. Finally, the impact of disorder on fear of crime is discriminatory, depending on the type and spatial structure. This study suggests that urban policy efforts focus more on indirect and macroscopic aspects in dealing with the decline of cities and managing issues related to social disorder.

## 1. Introduction

There has been a recent global increase in the incidence of various types of violent crimes, such as shootings and random murders, including in South Korea [1,2]. The public is continuously inundated with detailed reports of such crimes, resulting in growing uneasiness and fear within communities. Violent crimes not only enrage many people, but they trigger fears that they might become the next target. In fact, according to the National Survey on the People’s Living Safety (NSPLS) conducted by the Korean Institute of Criminology in 2014, 21.5% of Koreans harbor a fear of crime. Moreover, in regards to the awareness of trends in the occurrence of crime, 26.8% of the respondents believed that crime was increasing in their neighborhood, indicating that at least one of every five people are fearful of crime victimization. As such, fear of crime is prevalent in Korean society. The resulting psychological anxiety from such fear negatively impacts people’s lives. According to Stafford et al. [3], fear of crime has a negative impact on the mental health of individuals, limiting their physical and social abilities. Moreover, the prevalence of such fear in the neighborhood weakens the bonds between neighbors and the overall community network, thereby undermining social capital and impeding the city’s sustainability [4,5].

Accordingly, fear of crime is an important topic of research. In addition to individual characteristics such as gender, age, and income, fear of crime is closely linked to the physical and social characteristics of the neighborhood [6,7]. This relationship can be explained through the disorder phenomenon, in which disorder refers to the unfavorable physical or social conditions of a specific space (i.e., neighborhood or city). Disorder signifies that the control in the area is being undercut and that residents living in that area are in a state of social disorganization in which they cannot maintain effective social control by themselves [6,8]. Moreover, disorder promotes awareness that the area is dangerous, resulting in avoidance of that area and a reduction in its use, ultimately creating a vicious cycle of neglecting the disorderly state and behavior [9,10,11]. This cycle causes the area to decline or lag behind others, and residents feel that their neighborhood is vulnerable to crime regardless of objective levels of crime [5].

Numerous studies have demonstrated that disorder in a neighborhood has a significant impact on fear of crime [6,12,13,14,15,16,17,18]. However, disorder is a multilayered system that is dependent on the type (physical, social, subjective, and objective) and spatial level (neighborhood, city). The impacts of such structural characteristics on fear of crime are discriminative and complex. However, previous studies have failed to specifically examine the complex characteristics of disorder. Such analysis is important because it will enable the neighborhood and city to utilize their limited budget more effectively to implement urban policies. Additionally, because disorder generates a fear of crime even though actual crimes may not have occurred, its mere presence deteriorates the value of the entire community, accelerating its decline. Therefore, disorder constitutes a key factor hindering the sustainability of the city [10,19].

Addressing the need to examine the complex characteristics of disorder, this study examines fear of crime in Korea. Indeed, urban decline has recently emerged as a social problem in Korea. Residents are reluctant to live in declining or declined neighborhoods and cities with relatively disorderly environments because of the fear of crime. In response, the new-deal urban regeneration policy has been implemented to revitalize declined cities, with methods to reduce fear of crime suggested accordingly [20,21,22,23,24,25]. As such, contemporary, in-depth studies on disorder and fear of crime that emphasize the significance of the urban policy agenda are both necessary and timely.

This study classifies disorder on multiple levels: subjective or objective, based on how it is perceived, social or physical based on its tangible presence, and whether its spatial structure includes the neighborhood or wider community area, specifically at the city level. An empirical analysis using a multilevel model was conducted to determine how the multilayered characteristics of disorder affect fear of crime. Based on the results of this analysis, this study reviews the public policy agenda for a safe city in which quality of life and residential convenience is improved, and advances an in-depth understanding of the urban decline of cities undergoing or that are in danger of decline because of disorder and fear of crime. Finally, the implications of this study for the direction of urban policies is discussed, particularly in terms of enhancing urban sustainability and health.

## 2. Theoretical Background

In general, fear is defined as a normal reaction to a real or imagined threat [26,27]. As such, fear comprises an abstract concept as a phenomenon related to human emotions. In this respect, scholars vary on what fear of crime actually means and how it can be measured. For instance, Warr [28] understands fear of crime victimization as involving sensitivity to crimes, thus regarding it as an emotional response to danger and anxiety. This connotes that fear of crime does not have to be accompanied by injury or damage resulting from crime. Meanwhile, Garofalo [29] defines fear of crime as an emotional reaction characterized by a sense of danger and anxiety resulting from actual or perceived physical danger that is related to some aspect of crime. This understanding implies that fear of crime is an individual’s emotional response in an environmental condition where they perceive the possibility of crime occurring. Moreover, fear of crime is not a single feeling, but a complex of emotions and perceptions entwined in a complicated manner [30]. As such, it is necessary to distinguish fear of crime in order to determine its meaning. Broadly speaking, the vague fear one feels about crime or safety is referred to as generalized fear, while the fear evoked in assessing the possibility of becoming a victim of a certain crime is referred to as a specific fear [31].

Individual attributes serve as important factors in assessing fear of crime. According to the vulnerability theory, fear of crime victimization is determined by an individual’s perception of how much damage a crime would cause rather than the possibility of actually being victimized. The vulnerability of individuals indicates the degree to which they perceive themselves as potential victims of crime, and fear of crime victimization appears to be higher in groups with certain shared characteristics [29,32,33,34]. For instance, females constitute a group typically vulnerable to crime, and tend to have a greater fear of crime than males [35,36,37,38,39,40,41]. In this regard, Brown [42] analyzed fear of crime in Seoul, the capital of Korea, as well as in neighboring metropolitan areas. Brown categorized fear of crime into six types—including fear of home invasion or burglary, perceived risk of victimization during the day or night, and avoidance of nocturnal activity or particular areas due to fear of crime—and determined the factors influencing these types. In doing so, Brown demonstrated that gender was the only factor with a significant influence over all types of fear. Age is another important factor impacting fear of crime, with studies showing that adolescents and the elderly are more vulnerable and hence experience a higher fear of crime [43,44,45,46,47].

In terms of socio-economic status, Rountree [48] and Pantazis [49] have claimed that the low-income group exhibits a greater fear of crime than the high-income group. Indeed, several studies have demonstrated that such fear is greater among those with a lower income because they lack the ability to protect themselves from potential damage and tend to live in regions with higher crime rates [11,29,32,50,51,52,53]. However, Keane [31] argues that the high-income group exhibits a greater fear of crime because their physical assets (e.g., housing) are more likely to be the target of a crime. Some case studies conducted in Korea have also found that fear of crime increases with the level of income for similar reasons [54,55]. Meanwhile, other studies have shown that fear of crime is lower among whites and greater among racial groups [56,57], and among those with lower [53,56,57,58] or higher education levels [55,59,60].

Victimization theory is also concerned with the individual attributes that account for the fear of crime. Specifically, this theory explains that experience with direct or indirect damage is an important predictor of fear of crime [34]. Such experience has been shown to have a consistent adverse effect on fear of crime in the various demographic groups mentioned above. Several studies have emphasized that indirect experience is more important for fear of crime [61,62,63,64].

While vulnerability theory focuses on individual characteristics to explain fear of crime, community concern theory shows that community characteristics are closely related to fear of crime. In particular, the perception that the community level control mechanism is not working increases fear of crime. However, although factors affecting fear of crime among communities exist, residents are unafraid if there is a sense of trust that the community can effectively control them [11]. In this regard, several studies found that strong social ties and social control reduced fear of crime [7,9,65,66].

Disorder theory also explains that disorder in a neighborhood is closely related to fear of crime. This theory claims that fear of crime increases when residents become aware of the presence of disorder and understand it as a sign that social control is being destroyed in the area [7]. This neighborhood disorder can be classified into physical and social disorder [4,67]. The former refers to the way in which physical elements that are neglected, or not, and managed appropriately, tend to stand out, such as an abandoned lot or empty house, graffiti, and litter [10,68]. Meanwhile, social disorder is related to people’s behaviors, including their direct and indirect experiences of factors that disturb basic order, including public intoxication, homelessness, and juvenile delinquency [6,17,18,52,67]. As these disorders become more prominent, people recognize that their community is not under control [10]. This results in a sense that they might become victims of crime, leading to fear of crime.

Various studies on the impact of disorder on fear of crime have established and verified the correlation between disorder and fear of crime. For instance, McGarrell et al. [52] determined that factors affecting fear of crime include both demographic characteristics and the awareness of disorder in the neighborhood. More specifically, fear of crime tends to grow when visible signs of social disorder (e.g., people drinking in public spaces and drug addicts) and physical disorder (e.g., litter and noise pollution) are found in a neighborhood. Next, Gibson at al. [69] focused on social disorder and collective efficacy to explain fear of crime. They showed that social disorder had a negative impact on fear of crime, while collective efficacy reduced fear of crime [66]. Oh et al. [16] also investigated the impact of social disorder on fear of crime. As a result, they found that perceived social disorder, such as people openly selling and using illegal drugs, drunk drivers on the road, people drinking excessively in public, prostitutes visible on the streets, youth gangs, and vandalism, increases the fear of crime significantly.

Many studies found that in addition to perceived disorder or social disorder, the physical disorder surrounding a neighborhood has a negative influence on the fear of crime [6,13,14,18,70]. In this regard, Perkins and Taylor [17] have demonstrated that physical disorder is more often related to fear than social disorder. However, LaGrange et al. [67], who analyzed cities in the United States, and Lee [71], who analyzed cities in Korea, produced conflicting results, finding that social disorder has a greater impact on fear of crime than physical disorder. This indicates that the type of disorder impacting fear of crime may depend on the spatial level, such as country or region.

In terms of disorder theory, recent studies in Korea have predominantly comprised empirical analyses of Seoul and its vicinity—the country’s capital and most populated city. Roh et al. [72] have verified that perceived incivilities that may influence the quality of life, including both physical disorder (including dark areas and litter) and social disorder (including areas where juvenile delinquents tend to gather), have a direct impact on the fear and perceived risk of crime. They further claim that community policing does not have a statistically significant relationship with fear of crime. Analyzing the impact of Crime Prevention Through Environmental Design (CPTED) on fear of crime, Lee et al. [73] showed that applying CPTED that can successfully control physical and social disorder to the neighborhood is an effective means of reducing fear of crime. Cho and Park [74] have also verified the effects of disorder on fear of crime along with the demographic characteristics, number of closed-circuit televisions (CCTVs), and crime rates in Seoul. Their results indicate that disorder, such as litter, noise, violence, delinquency, and parking, has a significantly negative influence on fear of crime.

While previous theories emphasized disorder as the cause of fear of crime, social disorganization theory claims that the cause of fear of crime can also be explained by the structural characteristics of the community [75]. This theory examines crimes in a more macroscopic view, underscoring that the various characteristics of the communities surrounding humans—such as the structural, environmental, economic, and social characteristics—affect the occurrence or fear of crime. The first to demonstrate the relationship between the structural characteristics of a community and crime was Guerry [76] and Quetelet [77] who criticized contemporary studies that relied entirely on biological factors to explain the cause of crime. In doing so, they sought to examine crime using a macroscopic research methodology from the perspective of social structure. In the wake of such work, various studies used social disorganization theory to show that the low socio-economic status of a community, population migration, and complicated population structure serve to weaken the ability to control residents, thereby causing crime and disorder [8,56,78,79].

Related studies have produced some significant findings. First, studies have found that the economic structure of a community with high income inequality (income disparity) adds to residents’ fear of crime because they believe that public management is focused on areas of the community with a high income [58,80]. Meanwhile, Brunton-Smith and Sturgis [56] have revealed the effect of urbanization and population movement—which are related to community structure at the city level—on fear of crime victimization. More specifically, they show that when these elements change rapidly within a community, fear of crime victimization grows because of concern about increasing disorder. Meanwhile, Alda et al. [81] have determined how regional characteristics in developing countries affect fear of crime; their results indicated that fear of crime is greater in regions with more poverty, lower community cohesion, and lower awareness of police performance.

Studies specific to Korea have also been conducted. For instance, Lee and Holoviak [82] verified that labor market conditions from 1980 to 2001, particularly in terms of the unemployment rate for young men, were closely related to crime. Additionally, younger subjects were more prone to violent crimes, while older subjects were more likely to be involved in property crimes. Meanwhile, Roh et al. [83] determined which factors at the district level affect street and residential crime victimization. Variables at the district level included community disorder, community cohesion, poverty, residential mobility, the ratio of commercial land transactions over the total number of land transactions, and the ratio of adolescents between the ages of 15 and 19. The results reveal that poverty within a district is the only factor that affects street crime victimization, while the factors influencing residential crime victimization include community disorder, cohesion, and the ratio of adolescents between the ages of 15 and 19.

**Figure 1:**
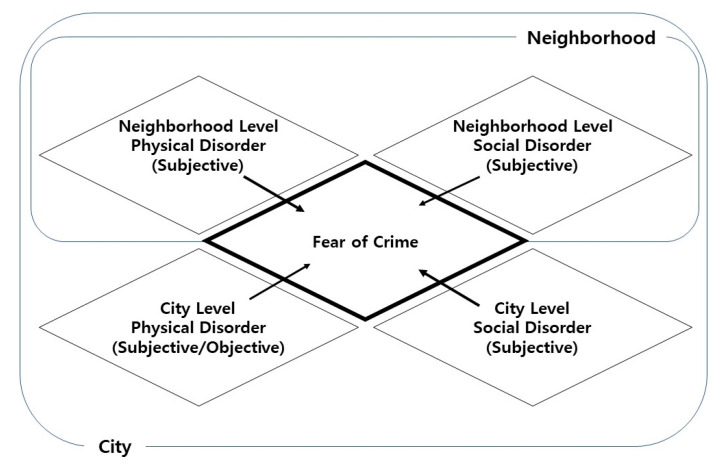
Multilayered relationship between disorder and fear of crime.

As such, the various characteristics of disorder have a significant influence on the fear of crime. However, as previously noted, disorder is produced through a multilayered process that is dependent on the type and spatial level. While these characteristics have a complex effect on the fear of crime, previous studies have failed to examine these relationships at the micro level. To overcome this limitation, this study includes a Korean national survey to determine disorder and individual fear of crime. Therefore, disorder is classified according to characteristics such as perception method (subjective or objective), physicality (physical or social), and spatial structure (neighborhood or city) (see Figure 1). The effect of such multilayered characteristics on fear of crime was then empirically analyzed. In doing so, this study addresses two gaps or weaknesses in the literature. First, it determines a more comprehensive cause for the fear of crime compared to previous studies. Second, it verifies the multilevel and complex effects of disorder that have been relatively overlooked in the existing literature. To this end, three key research hypotheses are established. First, disorder has a multilayered characteristic and affects the fear of crime. Second, subjective and objective disorder have different levels of effect on the fear of crime. Third, social and physical disorders have different levels of effect on the fear of crime.

## 3. Analytical Framework

### 3.1. Definition of Spatial Structure: Neighborhood and City

This study uses two spatial structures—neighborhood and city—to identify the effects of multilayered disorder characteristics on fear of crime. Therefore, defining spatial boundaries is a crucial step. In general, a neighborhood is recognized as a geographically localized area within a city or suburb [84]. However, there is little consensus on the definition of neighborhood. In the NSPLS, the neighborhood is defined as an area usually perceived by people as the main space of their lives. The neighborhood has similar housing price levels and shares schools, churches, hospitals, shopping places, and public transportation (bus or subway). The neighborhood described here is consistent with the concept of an area that enables interaction among neighbors based on the proximity of dwellings, and not on a clear boundary, as defined by Davies and Herbert [85] and Forrest and Kearns [86]. This study defines the neighborhood according to the NSPLS and uses it as a spatial boundary on the micro level (level 1).

In Korea, the city is a basic spatial unit that can coalesce statistical data and is usually given autonomy to efficiently carry out local administrative affairs. A city is referred to as “Si,” “Gun,” or “Gu,” respectively, depending on the population size and jurisdictions. Specifically, under the Local Autonomy Act in Korea (Article 7), local governments with a population greater than 50,000 are referred to as “Si,” while local governments installed in farming and fishing areas are “Gun.” In addition, local governments installed in the special cities of metropolitan and provincial governments are referred to as “Gu.” There are a total of 226 local governments in Korea, consisting of 75 “Si,” 82 “Gun,” and 69 “Gu.” Statistics reveal that the average population was 314,931 for “Si,” 53,462 for “Gun,” and 326,967 for “Gu” as of 2014. Data on the number of crimes committed in 2014, provided by Statistics of Korea, reveal a pattern similar to that of the population patterns. In Korea, cities, comprised of “Si,” “Gun,” and “Gu,” tend to be perceived as living spaces by residents. Moreover, most regional policies and research activities are also based on the city. In this regard, it is reasonable to consider the city (level 2) as an extended spatial structure of the neighborhood.

The broken windows theory states that disorder in the neighborhood is evidence of regional decline. Therefore, people get concerned that their city of residence is in decline based on neighborhood disorder [10,87]. Therefore, this study sets the spatial structure of the neighborhood to level 1 and that of the city to level 2 to examine the impact of multilayered disorder on fear of crime.

### 3.2. Data and Variables

This study investigates the multilayered characteristics of disorder and their impact on generalized fear of crime, rather than direct experiences of crime victimization. It thus utilizes the National Survey on the People’s Living Safety (NSPLS), which was conducted by the Korean Institute of Criminology, a national research center in Korea. The NSPLS is conducted every two years to determine Korean citizens’ perceptions of safety, as well as the crime victimization experiences of individuals and households in their daily lives. The government uses the results of these surveys as basic data in preventing crime and establishing protective policies for crime victims.

To determine how the multilayered disorder characteristics affect fear of crime, this study examined 14,722 individual responses on neighborhood perceptions as level 1 data. While there were 15,020 responses to the survey in 2014, the most recently disclosed data, incomplete surveys were excluded. City data is considered level 2 data and includes the disorder characteristics and the socio-economic index of the 217 cities where the survey was conducted.

For the dependent variable, this study used the response to the statement, “I am afraid of being a victim of crime,” which is a sub-item of the question, “How afraid are you about becoming a victim of crime in daily life?” The response to this statement was rated on a five-point Likert scale (1 = Not at all, 5 = Very much).

For the independent variables, this study included 18 variables associated with household characteristics, neighborhood risk, neighborhood social disorder (NSD), neighborhood physical disorder (NPD), city level social disorder (CSD), city level physical disorder (CPD), and socio-economic characteristics. More specifically, household characteristics include gender (SEX), age (AGE), level of education (EDUCATION), and monthly income (INCOME). Numerous studies have demonstrated that gender affects fear of crime in accordance with the vulnerability theory, and the general perception holds that women have a greater fear of crime. In terms of age, it is generally accepted that younger people have a greater fear of crime. The level of education was set as follows: 1 = elementary school or lower, 2 = middle school, 3 = high school, 4 = college (less than four years), 5 = university (four years), and 6 = graduate school or higher. Finally, income, which represents socio-economic status, also has a critical impact on fear of crime [31,48,49,54,55]. Therefore, monthly income is included in the variables. To maintain consistency with the survey, this study set the monthly income as follows: 1 = Less than KRW 1 million (approximately USD 880), 2 = KRW 1 million to less than KRW 2 million (approximately USD 880–1760), 3 = KRW 2 million to less than KRW 3 million (approximately USD 1760–2640), 4 = KRW 3 million to less than KRW 4 million (approximately USD 2640–3520), 5 = KRW 4 million to less than KRW 5 million (approximately USD 3520–4400), 6 = KRW 5 million to less than KRW 6 million (approximately USD 4400–5280), 7 = KRW 6 million to less than KRW 7 million (approximately USD 5280–6160), 8 = KRW 7 million to less than KRW 10 million (approximately USD 6160–8810), and 9 = KRW 10 million or more (approximately USD 8810 or more).

Regarding neighborhood risk, this study includes experience of crime victimization (VICTIM) and perception of policing in the neighborhood (POLICING). The NSPLS examines perception of policing based on three items: (1) the police are doing a good job in patrol activities, (2) the police seem like they would immediately get here if we call them to report a crime, and (3) the police seem like they would actually catch the criminal if we report a crime. The responses are based on a five-point Likert scale (1 = Strongly disagree, 5 = Strongly agree). This study uses the mean of the three items as the POLICING variable (see Table 1).

The neighborhood disorder characteristics used in this study predominantly comprise physical and social disorder. Physical and social disorders include awareness of the surrounding environment in the neighborhood based on six specific questions. These are sub-items of the NSPLS question, “How do you feel about the surrounding environment of your neighborhood?” This survey question constitutes an assessment of the neighborhood environment to which the respondent belongs, with residents providing their opinion on how socially and physically disorderly their neighborhood is. More specifically, items 1, 2, and 3 are the residents’ assessment of social disorder in the neighborhood (NSD); they include the violation of basic order, such as unauthorized crossing and illegal parking (BASIC), juvenile delinquents gathering in gangs (DELINQUENCY), and the witnessing of fights among residents (FIGHT). Items 4, 5, and 6 involve the assessment of physical disorder (NPD); they include the littering of waste (WASTE), dark and secluded spaces (SECLUD), and empty or abandoned buildings (EMPTY) (see Table 2).

Since the neighborhood level disorder used in this study was identified based on the NSPLS survey, a reliability test should be performed to determine internal consistency. This study used the Cronbach’s alpha test to verify internal consistency. The estimate of alpha will take on any value less than or equal to 1. A higher value of alpha implies higher reliability. A reliability of 0.6–0.7 is the acceptable threshold for survey data [88,89]. The test result showed a reliability of 0.785 for NSD and 0.784 for NPD, indicating that the characteristics of neighborhood level disorder used in this research are highly reliable.

According to the broken windows theory, all types of disorders that exist in a community may give people the impression that the area is neglected, resulting in a lack of regional management and the destruction of the area. The breakdown of order in a community accelerates the decline of the area [5,10]. City level disorder on such a broad scale is expected to have a negative impact on fear of crime, along with neighborhood level disorder. Decline related to city level disorder is caused by multiple and complex factors and is thus measured by various indicators. In Korea, the decline of a city is determined based on Article 17 of the Enforcement Decree of the Special Act on Promotion of and Support for Urban Regeneration. There are three criteria: namely, population and society, industrial economy, and residential environment. The city is considered to have declined when two or more of the criteria are met (see Table 3 for details). This decline is an objective index to determine how disorderly one’s city is. As such, this study set the state of city decline (DECLINE) as the index showing the objective level of physical disorder at the city level.

Additionally, the mean value of physical disorder (WASTE, SECLUD, EMPTY) in the neighborhood belonging to the city was used as an index showing the perception of city level physical disorder (CP_DISORDER). The mean value of social disorder (BASIC, DELINQUENCY, FIGHT) in the neighborhood belonging to the city was used as an index showing the perception of city level social disorder (CS_DISORDER).

Finally, to control the possible distortion of the statistical result due to the omitted variables, this study used city level socio-economic characteristics including local tax collections per capita in the city (TAX), population density in the city (DENSITY), and the number of crimes in the city per thousand people (CRIME) as the control variables. The use of these variables improved the model’s goodness of fit. Table 4 provides a description of the variables used in this study.

### 3.3. Methodology

To verify how disorder with a multilayered structure affects fear of crime, it is necessary to use independent variables that utilize level 1 data, which includes household and neighborhood characteristics, and level 2 data, which includes city characteristics. A multilevel model must be used because there is a multilevel structure between these two groups of variables. However, a general multilevel model that can suitably handle the multilevel data structure has two limitations: the dependent variables must be linear, and it must be assumed that the error term is in normal distribution at all levels. This implies that a general multilevel model is only applicable when the dependent variables are consecutive; if they are not, the link function must be applied [90]. Fear of crime—the dependent variable used in this study—is based on a five-point Likert scale, with higher points indicating greater fear. If the dependent variable has three or more items that are in order, a link function using cumulative probability is adopted to adequately handle them, and a multilevel ordered logistic model based on an ordered logistic model is used [91,92].

This study adopted the logistic model as the linking coefficient so that the dependent variable in Likert form can be interpreted in linear form. The link function of ordinal variables is shown in Equation (1). The link function is a logarithmic function that indicates how many times greater the probability is that the ordinal response $Y_{ij}$ is smaller than $y_{c}$, the *c*-th category of *Y*. By using this link function, cumulative probabilities $\eta_{mij}$ have a value in all real numbers. Therefore, the multilevel model predicting $\eta_{mij}$ can be used [91,93].

(1) $$\eta_{mij\;}\;=\;\log(\frac{\mathrm{Pr}\left(Y_{ij}\leq y_{c}\right)}{\mathrm{Pr}(Y_{ij}>y_{c})})$$

Analysis of the multilevel model is performed using an unconditional model that only includes constant terms and excludes explanatory (independent) variables. As shown in Equation (2), the unconditional model that comprises only level 1 and level 2 error terms ($\delta_{ij},\;\mu_{0j})$ only assumes the random effect of the constant terms and provides information to distinguish and compare the variance at each level. Here, constant term $\alpha_{00}$ refers to the mean of all samples [94].

(2) $$Y_{ij}\;=\;\alpha_{00}+\mu_{0j}+\delta_{ij}$$

Here, the calculated Intraclass Correlation Coefficient (ICC) refers to the variance explained by the difference between groups among the total variance of the dependent variable. This becomes the standard that shows how much the explanatory variables add to each level and explains the variance of the dependent variable [94,95]. Therefore, whether what is explained by level 2 variance is valid is determined by the ICC of the unconditional model. If the ICC is approximately 5–25%, the difference between multilevel groups is relatively large, which means that the multilevel model is valid. ICC can be presented in Equation (3) [91,96] as follows:

(3) $$\begin{array}{c} \mathrm{ICC}=\frac{\sigma_{u0}^{2}}{\sigma_{u0}^{2}+\sigma_{e}^{2}}\\ \sigma_{u0}^{2}:\;\mathrm{Level}\;2\;\mathrm{residual}\;\mathrm{variance}\;\mathrm{among}\;\mathrm{groups}\\ \sigma_{e}^{2}:\;\mathrm{Level}\;1\;\mathrm{residual}\;\mathrm{variance}\;\mathrm{among}\;\mathrm{individuals} \end{array}$$

After verifying the validity of the multilevel model through the unconditional model, a likelihood-ratio test was used to determine whether the existence of random effects is valid. The likelihood-ratio test is a method that determines whether random effects are statistically significant by comparing the model that considered the random effects and the model that did not. This study compared the case that includes only level 1 variables without considering the random effects with the full model, which also includes level 2 variables and considers the random effects. If the overall explanatory power of the model increases significantly when the random effects are considered compared to when they are not, the random intercept model in which the intercept is estimated differently depending on the characteristics of the high level is used. Here, it is assumed that the explanatory variable is a fixed coefficient and the intercept is a random coefficient. The level 1 and level 2 model can be presented, as shown in Equations (4) and (5) below [94]:

(4) $$\mathrm{Level}\;1\;\mathrm{Model}:\;Y_{ij}\;=\;\alpha_{00}+\sum_{p\;=\;1}^{P}\beta_{pj}X_{pij}+\mu_{0j}+\delta_{ij}$$

(5) $$\mathrm{Full}\;\mathrm{Model}:\;Y_{ij\;}\;=\;\alpha_{00}+\sum_{p\;=\;1}^{P}\beta_{pj}X_{pij}+\sum_{q\;=\;1}^{Qp}\beta_{0q}W_{qj}+\;\mu_{0j}+\delta_{ij}$$

In Formula (4), $Y_{ij}$ is the dependent variable that refers to the fear of crime of level 1 i that belongs to level 2 i. In the level 1 formula, $X_{pij}$ is the neighborhood level variable, which is the independent variable that predicts the dependent variable; this is the level 1 value in the order of i that belongs to level 2 city i. Here, $\beta_{pj}$ is the coefficient of $X_{pij}$, and $\alpha_{00}$ is the level 1 intercept. Moreover, $\delta_{ij}$ is the random effect of level 1 i that belongs to level 2 i, and the residual of fear of crime at the level 1 (neighborhood level). Next, $W_{pj}$ in Formula (5), which is the full model formula, is the variable at the level 2, and $u_{0j}$ is the residual of fear of crime at the level 2 (city level).

The results of the analysis are generally interpreted through the odds ratio that converted regression coefficient β into EXP(β). The odds ratio is a measure of association between an exposure and an outcome. The odds ratio represents the odds that an outcome will occur given a particular type of exposure, compared to the odds of that outcome occurring in the absence of that exposure [97]. In other words, the odds ratio is the increased ratio of the odds (probability of occurrence compared to probability of non-occurrence) of the dependent variable obtained when β of the independent variable increases by one unit, assuming that all values of other variables are constant and that the odds ratios have convenient interpretation in case-control studies [98].

In addition to empirical analysis using this multilevel model, this study verified the multilayered and complex effects of disorder. To achieve this, this study classified disorder by the perception method into subjective and objective disorder, by physicality (properties) into social and physical disorder, and by spatial structure into neighborhood and city disorder. The effects of such multilayered characteristics on fear of crime were then empirically analyzed. For such an analysis, this study verified whether there is a significant difference in fear of crime among different types based on the odds ratio of disorder variables that belong to the same type (group) using mean comparison, parametric tests (ANOVA, *t*-test), and non-parametric tests (Kruskal-Wallis, Mann-Whitney) depending on the homogeneity of variance and the number of samples.

## 4. Results

### 4.1. Descriptive Results

The results of the basic statistical analysis are reported in Table 5. The dependent variable FEAR had an average of 2.39, indicating that people have a certain generalized fear, but the fear of crime is not very high overall.

In the case of the household characteristics used as independent variables, the ratio of men and women was almost the same, while age was distributed between 14 and 98 years with a mean age of 48. The respondents were generally high school graduates, and their mean value of monthly income was 3.84, which falls within the category of between KRW 3 and 4 million (approximately USD 2640–3520).

Regarding neighborhood risk, few respondents (4%) had experience with direct damage (VICTIM) and felt that the police in their neighborhood were not engaged in active policing (POLICING). This indicates that there is a need for more reinforced policing, including patrols and quick mobilization in the case of a crime.

For neighborhood disorder, the perception that there were many people who do not keep basic order (BASIC) resulted in a mean of 2.42, the mean of the perception that there were many juvenile delinquents gathering in gangs (DELINQUENCY) was 2.14, and that of the perception that they could often see people fighting (FIGHT) was 2.12. This indicates that the respondents generally did not believe that there was much neighborhood social disorder (NSD). The negative perception of neighborhood physical disorder (NPD), such as excessive waste nearby (WASTE) or dark and secluded places (SECLUD), was comparatively higher than in the case of NSD. However, the negative perception that there are many empty and abandoned buildings (EMPTY) was the lowest. These results indicate that the perception of overall neighborhood level disorder was relatively low, which can be attributed to the fact that public management is conducted relatively strictly and thoroughly in Korea.

As for disorder in terms of city level, the perception that the city is in social disorder (CS_DISORDER) revealed a mean of 2.23, indicating that it is not overly negative. The perception of deterioration or the insufficient management of physical facilities in the city (CP_DISORDER) also showed a mean of 2.25, which is not very different from the perception of CS_DISORDER. However, the maximum values of CS_DISORDER and CP_DISORDER were 3.71 and 3.68, respectively, indicating that some cities were in a state of significant social or physical disorder. For DECLINE, which is an objective index that determines how physically disorderly an individual’s city is, the results reveal that 37% of the 217 cities examined in this study are in a state of decline.

For the socio-economic characteristics used to control possible statistical distortion resulting from the distinctive characteristics of individual cities, the local tax collections per capita (TAX) were KRW 930,000 on average (approximately USD 818) and the population density (DENSITY) averaged 5430 persons per km^2^. Finally, the number of crimes per thousand people (CRIME) was 35, but the city with the highest crime rate had at least ten times more crimes than that with the lowest crime rate, demonstrating that there is a relatively significant gap between Korean cities in terms of the risk of crime.

### 4.2. Empirical Analysis

For the empirical analysis, this study used an unconditional model to verify whether there are multilayered characteristics of disorder that affect fear of crime. The results indicate that the variance ratio explained by level 2 disorder characteristics was 26.18%, indicating that city level differences must be verified in order to explain the fear of crime. In other words, the multilayered disorder characteristics are significant in explaining fear of crime, and the multilevel model must thus be used. According to Lee and Noh [94], if the ratio of level 2 variance in total variance (ICC) is 5–25% in social science, the difference among level 2 groups is considered relatively large and the analysis using the multilevel model is proved to be valid. In this study, the level 2 ICC is over 26%, proving that there is sufficient need for the use of the multilevel model (see Table 6).

Prior to conducting empirical analysis, this study verified the validity of random effects through the likelihood-ratio test. The results indicate that the overall explanatory power of the model increases significantly when random effects are included at the significance level of 1%. Moreover, when level 2 variables were included, −2 Res Log Likelihood was smaller than the case in which only level 1 variables were used, proving that the full model including level 2 variables is more valid. Accordingly, the Akaike Information Criterion (AIC) and the Bayesian Information Criterion (BIC), which indicate the relative goodness-of-fit of the statistics used in this study, were also more suitable in the full model than the level 1 model. Thus, the results of analysis were explained with a focus on the full model.

The results of the empirical analysis show that all variables of individual characteristics, except EDUCATION and INCOME, affected fear of crime with statistical significance (see Table 7). As demonstrated by previous studies [35,36,37,38,39], women felt a greater fear of crime than men. As for age, older people tended to show less fear of crime since the majority of the older people do not live alone, and because people tend to think of age as an indicator of authority in Korea [99]. For education level and income, people with a higher education level and income exhibited a greater fear of crime, although there was no statistical significance. This is because highly educated groups obtain more information about various crimes, thus developing a greater fear of them. Additionally, people with high income believe that their wealth makes them a greater target [31] and that they will suffer from immense economic opportunity costs should they become victims of crime. In fact, as previously mentioned, case studies in Korea show that a higher income leads to a greater fear of crime because of similar reasons [54,55]. Moreover, those with experience of crime victimization have a greater fear of crime, and those with higher trust in and satisfaction with policing have less fear of crime.

Results regarding individuals’ perceptions of disorder in their neighborhood indicate that all variables related to social and physical disorder have a statistically significant influence on fear of crime. More specifically, in terms of NSD, respondents felt that they are not safe from crimes when there are many people who do not keep the basic order (BASIC), when there are many juvenile delinquents (DELINQUENCY), and when they often encounter people arguing or fighting loudly (FIGHT). Moreover, fear also tended to increase when respondents felt that there were numerous factors of physical disorder in the neighborhood. They felt their neighborhood was unsafe when their surroundings were dirty and littered (WASTE), when there were many dark and secluded places (SECLUD), and when there were many neglected cars or empty buildings (EMPTY).

In regard to the relationship between physical disorder and fear of crime at the city level, which is a higher and wider level than that of the neighborhood, CS_DISORDER has the greatest influence on fear of crime with an odds ratio (OR) of 2.961. Moreover, DECLINE that includes the objective phenomenon of physical disorder also adds to fear of crime. However, this study did not find any evidence that fear of crime is influenced by the perception of physical disorder (CP_DISORDER) at the extensive city level. This is because there is a difference in the intensity of fear as physical disorder is not discovered near the area in which individuals live. However, social disorder at the city level does not have a clear substance or scope; therefore, the thought that such disorder may occur near the area in which individuals live induces greater fear. In other words, when there are abandoned houses or significant litter far from the area in which respondents live, they can visually perceive and avoid the risk of crimes that may occur, making them less likely to fear crime as they feel that they can be safe if they are careful. On the other hand, if there is a rumor that a criminal who escaped from prison entered the city, the criminal’s radius of action is impossible to predict, and the residents thus feel a higher level of fear. The results of this study objectively explain this phenomenon, thus supporting the findings of other studies that social disorder has a greater effect on fear of crime than physical disorder [67,100]. Moreover, research conducted in Korea has similarly proved that social disorder has a greater effect on increasing the risk of crime victimization than physical disorder [71].

Of the variables related to city characteristics used as control variables, TAX and DENSITY had a statistically significant influence on fear of crime. TAX is an index representing individual wealth. As previously mentioned, people with higher income believe that their wealth makes them a target for crime and that they will suffer greater loss if victimized by crime, thereby inducing higher fear of crime. Moreover, a higher population density in the city increases complexity, which negatively affects the fear of crime.

To verify whether there is a significant difference in fear of crime among disorder type based on the odds, this study used parametric (including ANOVA and *t*-test) and non-parametric (including Kruskal-Wallis and Mann-Whitney) tests, as well as mean comparison to determine the effects of complex multilayered characteristics of disorder. Five models were constructed to verify the mean difference and multilevel effects (see Table 8 for details).

The first and second models were structured to examine the difference of influence according to spatial structure. In the multilevel comparison model, the types were categorized as neighborhood social disorder (NSD), neighborhood physical disorder (NPD), city social disorder (CSD), and city physical disorder (CPD) to determine the spatial and complex effects of disorder. This study then comparatively analyzed whether a significant difference in fear of crime existed among the different types using mean comparison, ANOVA, and Kruskal-Wallis. Next, in the neighborhood versus city model, mean comparison, *t*-test, and Mann-Whitney were used to examine the mean difference of influence according to spatial level in two types: neighborhood and city. The third and fourth models examined the influence according to the characteristics of disorder. The NSD versus NPD model determined the difference between social and physical disorder at the neighborhood level only using mean comparison, *t*-test, and Mann-Whitney. The CSD versus CPD model verified the difference between social disorder and physical disorder at the city level using the same methods. Finally, this study determined how the subjective perception and objective index of physical disorder show different effects on fear of crime using mean comparison, *t*-test, and Mann-Whitney. Here, the objective index of physical disorder includes DECLINE, as used by a Korean government agency to determine the physical decline of a city. Other variables based on the perception of respondents through the NSPLS were included in the category of subjective perception.

The results of the analyses reveal that there is a significant difference in the effects of multilevel comparison of disorder on fear of crime in each type. In particular, CSD had the greatest effect on fear of crime, followed by CPD. Neighborhood disorder, including NSD and NPD, had relatively little effect on fear of crime compared to disorder at the city level. In comparing disorder at the neighborhood and city level in detail, this study found that disorder at the city level had a greater effect on fear of crime. This result demonstrates that disorder at the city level, especially social disorder (CSD), is a more crucial factor in fear of crime than disorder at the neighborhood level. Comparing NPD and NSD in terms of physicality, this study found that the difference in the effect on fear of crime was not statistically significant. This indicates that whether the disorder is physical or social makes no difference in regard to fear of crime at the neighborhood level. However, at the city level, although the statistical significance could not be confirmed due to a lack of cases for comparison, social disorder (CSD) had a mean difference of 1.314 and was 80% higher than physical disorder (CPD). This implies that physicality of disorder tends to have a discriminatory effect on fear of crime depending on the spatial level. Finally, in comparing the influence between the subjective perception and objective index of disorder regardless of spatial level, this study found no evidence that objectively measured disorder or subjectively perceived disorder had a discriminatory effect on fear of crime. This is because the disorder subjectively perceived by individuals originates from an objective phenomenon.

In sum, this study empirically verified that disorder that can be subjectively perceived and disorder that objectively surrounds individuals at the neighborhood and city level both affect fear of crime. It also verified that the multilayered and complex characteristics of disorder have a discriminatory effect on fear of crime.

## 5. Discussion

A disorderly environment is perceived as causing or exacerbating the fear of crime among people, and as a problem hindering the sustainability of a city. Indeed, previous studies have demonstrated that neighborhood and city disorder have a significant influence on fear of crime. However, although disorder involves a multilayered process shaped by type and spatial scope, insufficient research has examined how these structural characteristics affect fear of crime at the microscopic level. Addressing this gap, this study used a multilevel ordered logistic model to empirically analyze how the multilayered characteristics of disorder affect fear of crime. In doing so, it sought a deeper understanding of cities undergoing or showing a risk of decline due to disorder and fear of crime.

In conducting empirical analyses, this study verifies the following results. First, as demonstrated in previous studies, individual characteristics have an immense effect on fear of crime. More specifically, gender is a crucial factor in fear of crime, with women showing relatively more fear than men. Moreover, younger people exhibit a greater fear of crime because people in Korea seldom live alone as they grow older, and because age is considered an indicator of authority. Income and fear of crime are also positively correlated because people with higher income are more likely to become a target of crime because of their wealth, and thus suffer greater opportunity costs [31].

Second, empirical analysis proves that people are relatively unaware of disorder occurring in their neighborhood, and more sensitive to disorder at the city level. This appears to be reflective of the Korean context. Korea’s social structure has traditionally been focused on the neighborhood rather than the city. There are rules for self-governance concerning mutual aid among members of a neighborhood (so-called “hyangyak” in Korean), and villages in which groups of relatives share the same last name and live together in a neighborhood are common. In this sense, the neighborhood is more familiar than the broader scope of the area (i.e., city), and the familiar community spirit of their neighborhood still remains. Thus, people have a higher tolerance for disorder that occurs in their neighborhood. Moreover, because of recent and rapid modernization, political, administrative, economic, and social problems are handled at the city level, with the city constituting a legal unit. Accordingly, people have come to understand disorder in a more macroscopic view.

Third, this study finds that social disorder has a more significant effect on fear of crime than physical disorder. Undergoing increasing economic development, the Korean government implemented an urban space modernization policy after the Korean War (1950–1953). This urban management system is well-organized in the public sector in Korean cities, resulting in relatively little physical disorder. Indeed, public transport in Korea is considered the cleanest in the world, and there are fewer physically underdeveloped areas, such as slums, in comparison to other countries. However, rapid economic growth has resulted in a dramatic increase in social disorder, such as the widening gap between the rich and poor, an increase in the number of the underprivileged, and the frequency of random crime. In this respect, disorder in Korea is viewed more seriously from the social perspective than from the physical perspective.

Fourth, this study confirms that fear of crime is affected more by indirect factors, such as rumors or signals, than by direct factors, such as a deteriorated physical element, and actual crimes. In particular, social disorder—including groundless and socially dangerous rumors—can be rapidly dispersed via social networks. As such, this study argues that it is necessary to first resolve issues that may cause social chaos before implementing direct policies, such as those directed toward the eradication of crime. For instance, groundless and biased information regarding people from certain countries was recently dispersed by several media outlets in Korea, triggering a fear of specific citizens and leading some people to avoid regions in which these people lived in groups. Indeed, such misinformation even generated occasional issues of discrimination [1,101].

Based on the empirical analysis, this study shows that the effect of disorder on fear of crime is discriminatory depending on type and spatial structure. In particular, fear of crime is affected more by the decline of the city than of the neighborhood with which individuals feel most familiar, and more by social disorder than physical disorder. This implies that the government’s policy efforts must focus more on indirect and macroscopic aspects in order to deal with the overall decline of cities and manage issues related to social disorder, rather than on implementing direct and microscopic management policies such as eliminating the physical disorder visible in specific areas or eradicating crime.

## 6. Conclusions

The conclusions of this study also imply that people have greater fear when there is more disorder caused by the perceived misbehavior of others. To solve this problem, it is necessary to establish ways to increase collective efficacy and social capital so that members of the community can control the behavior of individuals and groups in their community. In this respect, it may also be important to actively implement CPTED, which has yet to become generalized in Korea, in urban policies. This will more effectively improve the quality of life and promote the sustainability of the neighborhood and city.

Many countries in the world are facing urban problems similar to those of Korea, including city decline and socio-cultural conflict, and citizens feel that their safety is under threat. As improving the quality of life is essential to city sustainability, the conclusions of this study provide a general understanding of the direction for urban policies in terms of urban safety.

This study has some limitations, and based on these, improved research needs to be conducted in the future. While it was necessary to focus on individual cities because circumstances may vary within the same country, this study was unable to achieve this due to the lack of available data. Future studies should be able to determine more complex and nuanced results by collecting data from individual cities and conducting empirical analysis based on such data. Furthermore, by comparing the varying effects on the fear of crime between countries, the different perceptions of disorder can be examined more specifically and according to the unique context of each country, such as those where guns are legal or those with high crime rates. To this end, more data should be collected and developed based on international cooperation. Finally, it is possible to identify the micro-effect of spatial characteristics on the fear of crime by subdividing the spatial boundary, such as the immediate neighborhood or adjacent houses.

## Figures and Tables

**Table 1:** Questions on perception of neighborhood policing.

Question	Unit
(1) The police are doing a good job in patrol activities	1 = Strongly Disagree 2 = Disagree 3 = Average 4 = Agree 5 = Strongly Agree
(2) The police seem like they would immediately get here if we call them to report a crime	
(3) The police seem like they would actually catch the criminal if we report a crime	

**Table 2:** Question of neighborhood level disorder perception.

How do You Feel About the Surrounding Environment of Your Neighborhood?				
Question	Variable	Category	Cronbach’s Alpha	Unit
(1) Violation of basic order?	BASIC	NSD	0.785	1 = Strongly Disagree 2 = Disagree 3 = Average 4 = Agree 5 = Strongly Agree
(2) Juvenile delinquents gathering in gangs?	DELINQUENCY			
(3) Witnessing fights among residents?	FIGHT			
(4) Littering of waste?	WASTE	NPD	0.784	
(5) Dark and secluded spaces?	SECLUD			
(6) Empty, abandoned buildings?	EMPTY			

NSD = Neighborhood Social Disorder; NPD = Neighborhood Physical Disorder.

**Table 3:** Estimation method for city decline.

Criteria	Estimation Method
Population and Society	(1) A city where the current population has decreased by more than 20% compared to the time when the population was the highest in 30 years, or (2) A city that has experienced a decline in population over three consecutive years in the last five years
Industrial Economy	(1) A city where the total number of businesses has decreased by more than 5% compared to the time when the total number of businesses was the highest in the last ten years, or (2) A city where the total number of businesses has decreased over three consecutive years in the last five years
Residential Environment	A city where over 50% of buildings were constructed more than 20 years ago

Source: Article 17 of the Enforcement Decree of the Special Act on Promotion of and Support for Urban Regeneration.

**Table 4:** Description of variables.

Variable		Description	Unit
Dependent Variable	FEAR	Generalized fear of crime	1 = Not at all, 2 = A little afraid, 3 = Somewhat afraid, 4 = Quite afraid, 5 = Very afraid
Level 1			
Household Characteristics	SEX	Gender	1 = Man, 0 = Woman
	AGE	Year of age	Year
	EDUCATION	Level of education	1 = Elementary or below, 2 = Middle, 3 = High, 4 = College (2 years), 5 = University (4 years), 6 = Graduate or higher
	INCOME	Average monthly income	1 = Less than KRW 1 million, 2 = KRW 1–2 million, 3 = KRW 2–3 million, 4 = KRW 3–4 million, 5 = KRW 4–5 million, 6 = KRW 5–6 million, 7 = KRW 6–7 million, 8 = KRW 7–10 million, 9 = More than KRW 10 million
Neighborhood Risk	VICTIM	Crime victimization experience	1 = If experienced, 0 = None
	POLICING	Perception about policing in the neighborhood	1 = Very negative, 2 = Negative, 3 = Average, 4 = Positive, 5 = Very positive
Neighborhood Social Disorder (NSD)	BASIC	Violation of basic order	1 = Strongly Disagree, 2 = Disagree, 3 = Average, 4 = Agree, 5 = Strongly Agree
	DELINQUENCY	Juvenile delinquents gathering in gangs	
	FIGHT	Witnessing of fights among residents	
Neighborhood Physical Disorder (NPD)	WASTE	Littering of waste	
	SECLUD	Dark and secluded space	
	EMPTY	Empty, abandoned buildings	
Level 2			
City Level Social Disorder (CSD)	CS_DISORDER	Perception of social disorder in the city	Number
City Level Physical Disorder (CPD)	CP_DISORDER	Perception of physical disorder in the city	Number
	DECLINE	State of city’s decline	1 = If declined, 0 = Other
Socio-economic Characteristics	TAX	Local tax collection per capita in the city	KRW 10,000
	DENSITY	Population density in the city	Thousand people/km^2^
	CRIME	Number of crimes in the city	Thousand people

**Table 5:** Results of descriptive statistics.

Variables		Mean	St. Dev.	Min	Max
Dependent	FEAR	2.39	1.11	1.00	5.00
Household Characteristics	SEX	1.53	0.50	1.00	2.00
	AGE	48.38	18.37	14.00	98.00
	EDUCATION	3.00	1.33	1.00	6.00
	INCOME	3.82	1.86	1.00	9.00
Neighborhood Risk	VICTIM	0.04	0.18	0.00	1.00
	POLICING	3.40	0.71	1.00	5.00
NSD	BASIC	2.42	0.97	1.00	5.00
	DELINQUENCY	2.14	0.87	1.00	5.00
	FIGHT	2.12	0.85	1.00	5.00
NPD	WASTE	2.35	0.93	1.00	5.00
	SECLUD	2.34	0.98	1.00	5.00
	EMPTY	2.06	0.90	1.00	5.00
CSD	CS_DISORDER	2.23	0.38	1.02	3.71
CPD	CP_DISORDER	2.25	0.38	1.21	3.68
	DECLINE	0.37	0.48	0.00	1.00
Socio-economic Characteristics	TAX	92.93	63.20	0.06	811.46
	DENSITY	5.43	6.51	0.02	27.94
	CRIME	34.98	11.78	11.06	114.17

**Table 6:** Result of unconditional model analysis.

Level	Unconditional Model	ICC
Level 1	0.9661	0.7382 (73.82%)
Level 2	0.3426	0.2618 (26.18%)
Total Variation	1.3087	1.00 (100%)

ICC = Intraclass Correlation Coefficient.

**Table 7:** Results of empirical analysis.

Variable		Level 1 Model			Full Model			VIF
		Estimate	OR	*t*-Value	Estimate	OR	*t*-Value	
Intercept5		−7.888 ***	-	−34.34	−10.359 ***	-	−22.17	-
Intercept4		−4.747 ***	-	−21.74	−7.212 ***	-	−15.64	-
Intercept3		−3.385 ***	-	−15.61	−5.852 ***	-	−12.72	-
Intercept2		−1.597 ***	-	−7.41	−4.067 ***	-	−8.86	-
Level 1 (*n* = 14,722)								
Household Characteristics	SEX	1.134 ***	3.107	34.92	1.126 ***	3.082	34.70	1.01
	AGE	−0.011 ***	0.989	−11.29	−0.011 ***	0.989	−11.37	1.22
	EDUCATION	0.125	1.133	2.33	0.129	1.138	1.45	1.10
	INCOME	0.005	1.005	0.74	0.003	1.003	0.45	1.19
Neighborhood Risk	VICTIM	0.378 ***	1.459	4.41	0.384 ***	1.467	4.49	1.01
	POLICING	−0.054 **	0.947	−2.09	−0.065 **	0937	−2.51	1.07
NSD	BASIC	0.068 ***	1.071	3.10	0.060 ***	1.062	2.73	1.68
	DELINQUENCY	0.138 ***	1.148	5.05	0.130 ***	1.139	4.76	2.15
	FIGHT	0.124 ***	1.132	4.36	0.114 ***	1.121	4.02	2.15
NPD	WASTE	0.079 ***	1.082	3.29	0.077 ***	1.080	3.19	1.89
	SECLUD	0.178 ***	1.195	7.62	0.177 ***	1.193	7.55	2.04
	EMPTY	0.066 ***	1.069	2.82	0.064 ***	1.066	2.72	1.68
Level 2 (*n* = 217)								
CSD	CS_DISORDER	-	-	-	1.086 ***	2.961	4.79	3.14
CPD	CP_DISORDER	-	-	-	0.587	1.798	2.80	2.94
	DECLINE	-	-	-	0.402 ***	1.495	2.71	1.24
Socio-economic Characteristics	TAX	-	-	-	0.109 *	1.115	1.46	1.35
	DENSITY	-	-	-	0.017 *	1.017	1.36	1.41
	CRIME	-	-	-	0.012	1.012	0.18	1.34
−2 Res Log Likelihood		36,707.18			36,628.31			-
AIC		36,741.18			36,676.31			-
BIC		36,798.64			36,757.43			-
Likelihood-Ratio (LR) Test		LR chi^2^(6) = 5.71 Prob > 0.007						

Note: * *p* < 0.1, ** *p* < 0.05, *** *p* < 0.01; OR = Odds Ratio.

**Table 8:** Multilayered disorder impacts on the fear of crime.

Type	Variable	Odds Ratio	Spatial Structure						Physicality						Perception Method		
			Multilevel Comparison			Neighborhood vs. City			NSD vs. NPD			CSD vs. CPD			Subjective vs. Objective		
			Mean	ANOVA	Kruskal-Wallis	Mean	*t*-Test	Mann-Whitney	Mean	*t*-Test	Mann-Whitney	Mean	*t*-Test	Mann-Whitney	Mean	*t*-Test	Mann-Whitney
NSD	BASIC	1.062	1.107	86.418 ***	5.622	1.110	10.98 **	−2.324 **	1.107	−0.122	−0.218	-			1.428	−0.096	−0.775
	DELINQUENCY	1.139															
	FIGHT	1.121															
NPD	WASTE	1.080	1.113						1.113								
	SECLUD	1.193															
	EMPTY	1.066															
CSD	CS_DISORDER	2.961	2.961			2.085			-			2.961	5.009	−1.225			
CPD	CP_DISORDER	1.798	1.647									1.647					
	DECLINE	1.495													1.495		

Note 1: CP_DISORDER is not statistically significant at the 10% level. Note 2: ** *p* < 0.05, *** *p* < 0.0.

## References

[1] Kang S., Seo W. (2018). The effect of nationality of foreign residents on fear of crime for local resident. J. Reg. Stud. Dev..

[2] Mancik A.M., Hawk S.R., Jarvis J.P., Regoeczi W.C. (2000). Evaluating fluctuations in homicide: Crowdsourcing trends and assessing sentiments of change. J. Crime Justice.

[3] Stafford M., Chandola T., Marmot M. (2007). Association between fear of crime and mental health and physical functioning. Am. J. Public Health.

[4] Skogan W.G. (1990). Disorder and Decline: Crime and the Spiral of Decay in American Neighborhoods.

[5] Wilson J.Q., Kelling G.L. (1982). Broken windows. Atl. Mon..

[6] Covington J., Taylor R.B. (1991). Fear of crime in urban residential neighborhoods: Implications of between-and within-neighborhood sources for current models. Sociol. Q..

[7] Lewis D.A., Salem G.W. (1986). Fear of Crime: Incivility and the Production of a Social Problem.

[8] Sampson R.J., Groves W.B. (1989). Community structure and crime: Testing social-disorganization theory. Am. J. Sociol..

[9] Rountree P.W., Land K.C. (1996). Perceived risk versus fear of crime: Empirical evidence of conceptually distinct reactions in survey data. Soc. Forces.

[10] Seo W., Von Rabenau B. (2011). Spatial impacts of microneighborhood physical disorder on property resale values in Columbus, Ohio. J. Urban Plan. Dev..

[11] Taylor R.B., Hale M. (1986). Testing alternative models of fear of crime. J. Crim. Law Criminol..

[12] Hardyns W., Pauwels L.J., Heylen B. (2018). Within-individual change in social support, perceived collective efficacy, perceived disorder and fear of crime: Results from a two-wave panel study. Br. J. Criminol..

[13] Hinkle J.C. (2013). The relationship between disorder, perceived risk, and collective efficacy: A look into the indirect pathways of the broken windows thesis. Crim. Justice Stud..

[14] Innes M. (2004). Signal crimes and signal disorders: Notes on deviance as communicative action. Br. J. Sociol..

[15] McCrea R., Shyy T.K., Western J., Stimson R.J. (2005). Fear of crime in Brisbane: Individual, social and neighbourhood factors in perspective. J. Sociol..

[16] Oh G., Ren L., He P. (2019). Social disorder and residence-based fear of crime: The differential mediating effects of police effectiveness. J. Crim. Justice.

[17] Perkins D.D., Taylor R.B. (1996). Ecological assessments of community disorder: Their relationship to fear of crime and theoretical implications. Am. J. Commun. Psychol..

[18] Wyant B.R. (2008). Multilevel impacts of perceived incivilities and perceptions of crime risk on fear of crime: Isolating endogenous impacts. J. Res. Crime Delinq..

[19] Han S. (2020). Compositional and Contextual Associations of Social Capital and Fear of Crime. Deviant Behav..

[20] An H., Song A., Park J., Yun H. (2019). A qualitative analysis of the urban regeneration priority projects: Focused on eleven neighborhood-based regeneration areas. J. Korea Plan. Assoc..

[21] Choi Y.J., Lee J.L. (2016). A study on the impact of the safe village residents’ awareness of CPTED on their fear of crime. Korean Assoc. Police Sci. Rev..

[22] Jang C. (2020). The influence of urban regeneration project on place attachment and settlement consciousness: Focused on urban vitality development project in Daegu. J. Korea Plan. Assoc..

[23] Jeong Y., Kang Y., Lee M. (2017). Effectiveness of a project Applying Crime Prevention through Environmental Design in an Urban Area in South Korea. J. Asian Arch. Build..

[24] Lee C.H., Choi J.H., Yun W.S. (2016). Effects of CPTED related COP activities on social capital, community disorder, and fear of crime victimization: A longitudinal analysis. J. Community Saf. Secur. Environ. Des..

[25] Park H. (2010). Designing out crime in South Korea: Qualitative analysis of contemporary CPTED-related issues. Asia Pac. J. Police Crim. Justice.

[26] Burnham J.J., Gullone E. (1997). The fear survey schedule for children II: A psychometric investigation with American data. Behav. Res..

[27] Kagan J., Schulkin J. (1995). On the concepts of fear. Harv. Rev. Psychiatry.

[28] Warr M. (2000). Fear of crime in the United States: Avenues for research and policy. Crim. Justice.

[29] Garofalo J. (1981). The fear of crime: Causes and consequences. J. Crim. Law Crim..

[30] Hough M. (1995). Anxiety about Crime: Findings from the 1994 British Crime Survey.

[31] Keane C. (1992). Fear of crime in Canada: An examination of concrete and formless fear of victimization. Can. J. Criminol..

[32] Baumer T.L. (1985). Testing a general model of fear of crime: Data from a national sample. J. Res. Crime Delinq..

[33] Killias M., Clerici C. (2000). Different measures of vulnerability in their relation to different dimensions of fear of crime. Br. J. Criminol..

[34] Skogan W.G., Maxfield M.G. (1981). Coping with Crime: Individual and Neighborhood Reactions.

[35] Boateng F.D., Adjekum-Boateng N.S. (2017). Differential perceptions of fear of crime among college students: The race factor. J. Ethn. Crim. Justice.

[36] Clemente F., Kleiman M.B. (1977). Fear of crime in the United States: A multivariate analysis. Soc. Forces.

[37] Crossman J.S., Rader N.E. (2011). Fear of crime and personal vulnerability: Examining self-reported health. Sociol. Spectr..

[38] Ferraro K.F. (1995). Fear of Crime: Interpreting Victimization Risk.

[39] Schafer J.A., Huebner B.M., Bynum T.S. (2006). Fear of crime and criminal victimization: Gender-based contrasts. J. Crim. Justice.

[40] Snedker K.A. (2015). Neighborhood conditions and fear of crime: A reconsideration of sex differences. Crime Delinq..

[41] Macassa G., Winersjo R., Wijk K., McGrath C., Ahmadi N., Soares J. (2017). Fear of crime and its relationship to self-reported health and stress among men. J. Public Health Res..

[42] Brown B. (2016). Fear of crime in South Korea. Int. J. Crime Justice Soc. Democr..

[43] Lagrange R.L., Ferraro K.F. (1987). The elderly’s fear of crime: A critical examination of the research. Res. Aging.

[44] Lee G.R. (1983). Social integration and fear of crime among older persons. J. Gerontol..

[45] May D.C. (2001). The effect of fear of sexual victimization on adolescent fear of crime. Sociol. Spectr..

[46] Pain R. (2001). Gender, race, age and fear in the city. Urban Stud..

[47] Warr M. (1984). Fear of victimization: Why are women and the elderly more afraid?. Soc. Sci. Q..

[48] Rountree P.A. (1998). A Reexamination of the crime-fear linkage. J. Res. Crime Delinq..

[49] Pantazis C. (2000). Fear of crime, vulnerability and poverty. Br. J. Criminol..

[50] Adams R.E., Serpe R.T. (2000). Social integration, fear of crime, and life satisfaction. Sociol. Perspect..

[51] Bennett R.R., Flavin J.M. (1994). Determinants of fear of crime: The effect of cultural setting. Justice Q..

[52] McGarrell E.F., Giacomazzi A.L., Thurman Q.C. (1997). Neighborhood disorder, integration, and the fear of crime. Justice Q..

[53] Will J., McGrath J. (1995). Crime, neighborhood perceptions, and the underclass: The relationship between fear of crime and class position. J. Crim. Justice.

[54] Park J. (2011). The community effect on the fear of crime. Korean J. Crime Delinq..

[55] Park J., Lee S. (2010). A multi-level approach to fear of crime: Testing four major models. Korean Criminol. Rev..

[56] Brunton-Smith I., Sturgis P. (2011). Do neighborhoods generate fear of crime? An empirical test using the British crime survey. Criminology.

[57] Franklin T.W., Franklin C.A., Fearn N.E. (2008). A multilevel analysis of the vulnerability, disorder, and social integration models of fear of crime. Soc. Justice Res..

[58] Vauclair C., Bratanova B. (2017). Income inequality and fear of crime across the European Region. Eur. J. Criminol..

[59] Lee M., Ulmer J. (2000). Fear of crime among Korean Americans in Chicago communities. Criminology.

[60] Paek S., Nalla M. (2015). The relationship between receiving phishing attempt and identity theft victimization in South Korea. Int. J. Law Crime Justice.

[61] Heath L., Petraitis J. (1987). Television viewing and fear of crime: Where is the mean world?. Basic Appl. Soc. Psychol..

[62] Intravia J., Wolff K.T., Paez R., Gibbs B.R. (2017). Investigating the relationship between social media consumption and fear of crime: A partial analysis of mostly young adults. Comput. Hum. Behav..

[63] Liska A.E., Baccaglini W. (1990). Feeling safe by comparison: Crime in the newspapers. Soc. Probl..

[64] Williams P., Dickinson J. (1993). Fear of crime: Read all about it? The relationship between newspaper crime reporting and fear of crime. Br. J. Criminol..

[65] Markowitz F.E., Bellair P.E., Liska A.E., Liu J. (2001). Extending social disorganization theory: Modeling the relationships between cohesion, disorder, and fear. Criminology.

[66] Yuan Y., McNeeley S. (2017). Social ties, collective efficacy, and crime-specific fear in Seattle neighborhood. Vict. Offenders.

[67] LaGrange R.L., Ferraro K.F., Supancic M. (1992). Perceived risk and fear of crime: Role of social and physical incivilities. J. Res. Crime Delinq..

[68] Seo W. (2018). Does neighborhood condition create a discount effect on house list prices? Evidence from physical disorder. J. Real Estate Res..

[69] Gibson C.L., Zhao J., Lovrich N.P., Gaffney M.J. (2002). Social integration, individual perceptions of collective efficacy, and fear of crime in three cities. Justice Q..

[70] Bolger M.A., Bolger P.C. (2019). Predicting fear of crime: Results from a community survey of a small city. Am. J. Crim. Justice.

[71] Lee S. (2000). Neighborhood factors and fear of crime: Testing a risk interpretation model. Korean Criminol. Rev..

[72] Roh S., Kwak D.H., Kim E. (2013). Community policing and fear of crime in Seoul: A test of competing models. Policing.

[73] Lee J., Park S., Jung S. (2016). Effect of crime prevention through environmental design (CPTED) measures on active living and fear of crime. Sustainability.

[74] Cho J.T., Park J. (2017). Exploring the effects of CCTV upon fear of crime: A multi-level approach in Seoul. Int. J. Law Crime Justice.

[75] Lilly J.R., Cullen F.T., Ball R.A. (2019). Criminological Theory: Context and Consequences.

[76] Guerry A.M. (1833). Essai sur la Statistique Morale de la France.

[77] Quetelet A. (1835). Sur l’Homme et le Développement de ses Facultés ou Essai de Physique Sociale.

[78] Grasmick H.G., Tittle C.R., Bursik R.J., Arneklev B.J. (1993). Testing the core empirical implications of Gottfredson and Hirschi’s general theory of crime. J. Res. Crime Delinq..

[79] Sampson R.J., Raudenbush S.W., Earls F. (1997). Neighborhoods and violent crime: A multilevel study of collective efficacy. Science.

[80] Hummelsheim D., Hirtenlehner H., Jackson J., Oberwittler D. (2011). Social insecurities and fear of crime: A cross-national study on the impact of welfare state policies on crime-related anxieties. Eur. Sociol. Rev..

[81] Alda E., Bennett R.R., Morabito M.S. (2017). Confidence in the police and the fear of crime in the developing world. Policing.

[82] Lee D.Y., Holoviak S.J. (2006). Unemployment and crime: An empirical investigation. Appl. Econ. Lett..

[83] Roh S., Kim E., Yun M. (2010). Criminal victimization in South Korea: A multilevel approach. J. Crim. Justice.

[84] Pickett K.E., Pearl M. (2001). Multilevel analyses of neighborhood socioeconomic context and health outcomes: A critical review. J. Epidemiol. Commun. Health.

[85] Davies W.K.D., Herbert D.T. (1993). Communities within Cities: An Urban Social Geography.

[86] Forrest R., Kearns A. (2001). Social cohesion, social capital and the neighbourhood. Urban Stud..

[87] Ross C.E., Mirowsky J. (1999). Disorder and decay: The concept and measurement of perceived neighborhood disorder. Urban Aff. Rev..

[88] Nunnally J.C. (1978). Psychometric Theory.

[89] Peterson R.A. (1994). A meta-analysis of Cronbach’s coefficient alpha. J. Consum. Res..

[90] Raudenbush S.W., Bryk A.S. (2002). Hierarchical Linear Models: Applications and Data Analysis Methods.

[91] Grilli L., Rampichini C., Kenett R., Salini S. (2012). Multilevel models for ordinal data. Modern Analysis of Customer Surveys: With Applications Using R.

[92] O’Connell A.A. An illustration of multilevel models for ordinal response data. Data and context in statistics education: Towards an evidence-based society. Proceedings of the Eighth International Conference on Teaching Statistics.

[93] Kang S. (2016). Multilevel Models.

[94] Lee H., Noh S. (2013). Advanced Statistical Analysis: Theory and Practice.

[95] Lorah J. (2018). Effect size measures for multilevel models: Definition, interpretation, and TIMSS example. Large Scale Assess. Educ..

[96] Bauer D., Sterba S. (2011). Fitting multilevel models with ordinal outcomes: Performance of alternative specifications and methods of estimation. Psychol. Methods.

[97] Szumilas M. (2010). Explaining odds ratios. J. Can. Acad. Child Adolesc. Psychiatry.

[98] Glas A.S., Lijmer J.G., Prins M.H., Bonsel G.J., Bossuyt P.M. (2003). The diagnostic odds ratio: A single indicator of test performance. J. Clin. Epidemiol..

[99] Chang A.S., Jung H.W., Park C.H. (2011). Victimization-fear paradox in Korea: An interactive model with age from a developmental and life-course perspective. Korean Criminol. Rev..

[100] Yates A., Ceccato V. (2020). Individual and spatial dimensions of women’s fear of crime: A Scandinavian study case. Int. J. Comp. Appl. Crim. Justice.

[101] Im Y., Cho Y., Kim E. (2017). Portrayals of foreign-born residents in South Korean crime news. Korea Obs..

